# 289. Evaluation of Aztreonam-Avibactam I*n Vitro A*ctivity Against VIM and GES β-lactamase Producing, Carbapenem-Resistant *Pseudomonas aeruginosa* Collected from Contaminated Artificial Tear Products

**DOI:** 10.1093/ofid/ofad500.361

**Published:** 2023-11-27

**Authors:** Lynn-Yao Lin, Dmitri Debabov

**Affiliations:** Abbvie, Irvine, California; Abbvie, Irvine, California

## Abstract

**Background:**

A rare strain of extensively drug-resistant *Pseudomonas aeruginosa* was identified from contaminated artificial tear products that caused outbreak across 16 US states in March 2023. The strain carries Verona integron-encoded metallo-β-lactamase (VIM) and Guiana-Extended Spectrum-β-Lactamase (GES), a combination not previously identified in the US. Aztreonam (ATM) is a monobactam that is stable against hydrolysis by MBLs, and avibactam (AVI) inhibits class A, C, and some D β-lactamases. ATM-AVI is currently in development for treatment of infections caused by drug-resistant Gram-negative pathogens, especially those co-producing MBLs and other β-lactamases. This study evaluated *in vitro* activity of ATM-AVI against VIM and GES producing carbapenem-resistant *P. aeruginosa* (VIM-GES-CRPA) isolates acquired from CDC AR Bank.

**Methods:**

Three VIM-GES-CRPA clinical isolates acquired from CDC AR Bank were tested for susceptibility to ATM and ATM-AVI (fixed concentration of 4 mg/L AVI) by broth microdilution (BMD). The isolates were tested concurrently by disk diffusion method using ATM-AVI 30-20 µg disks manufactured by BD Biosciences (BD) and Mast Group (Mast), as well as cefiderocol and ATM disks by Hardy diagnostics (Hardy) and by ATM-AVI E-test strips by Liofilchem, Inc. β-lactam/β-lactamase inhibitors (BL/BLI) were tested by BMD. All tests were interpreted using CLSI 2023 breakpoints. Tentative ATM-AVI pharmacokinetic/pharmacodynamic (PK/PD) susceptible MIC breakpoints of ≤ 8 mg/L (BMD, E-test) or ≥ 23 mm (disk diffusion) were applied for comparison purposes.

**Results:**

The MIC values of ATM-AVI by BMD were in the range of 4-8 mg/L and correlated with the values measured by ATM-AVI E-test (3-8 mg/L), demonstrating in vitro activity against VIM-GES-CRPA. ATM-AVI inhibition zone diameters measured by both BD and Mast discs were in the range of 27-28 mm. The isolates were shown to be resistant to ATM alone, ceftazidime/avibactam, ceftolozane/tazobactam and imipenem/relebactam, but susceptible to cefiderocol.

**Conclusion:**

Based on proposed PK/PD breakpoints, all tested isolates of VIM-GES-CRPA were susceptible to ATM-AVI. ATM-AVI demonstrated in vitro activity against these extensively drug-resistant *P. aeruginosa* isolated from contaminated artificial tear products.

**Disclosures:**

**Lynn-Yao Lin, M.S. Molecular Biotechnology**, Abbvie Inc: Employee of Abbvie that participated in design, study conduct, financial support, interpretation of data, review, and approval of the publication **Dmitri Debabov, PhD. Molecular biology**, Abbvie Inc.: Employee of Abbvie that participated in design, study conduct, financial support, interpretation of data, review, and approval of the publication

